# Health Professions Educational Debt: Personal, Professional, and Psychological Impacts 5 Years Post-graduation

**DOI:** 10.3389/fmed.2022.746463

**Published:** 2022-02-10

**Authors:** Patrick Webster, Sara E. North

**Affiliations:** Division of Physical Therapy, University of Minnesota, Minneapolis, MN, United States

**Keywords:** education, debt, impact, health professions, physical therapy

## Abstract

**Introduction:**

Cost burden in health professions education is rising. To bridge the gap between growing tuition and stagnating wages, student loans are increasingly obtained to cover educational costs. The spiraling after-effects are a source of acute concern, raising alarms across institutions and occupations. There is little dissemination to date of feasible data collection strategies and outcomes beyond 1 year post-graduation. Research is needed in evaluating the impacts of healthcare educational debt on career and personal choices following transition to practice.

**Materials and Methods:**

This study utilized a cross-sectional, mixed methods design. Doctor of Physical Therapy (DPT) Program graduates 5 years following degree completion completed a quantitative online survey, with topics including debt-to-income ratio, educational debt repayment strategies, impact on personal factors, non-education debt, and perceived value of their health professions education. Subsequent phone interviews were conducted by student researchers to gain insights into alumni perceptions of the impacts of educational debt on personal and professional decision-making. Data analysis involved descriptive and correlational quantitative statistics and open and axial coding of interview constructs.

**Results:**

The mixed methods format was successful in obtaining desired depth of response data. Quantitative findings demonstrated primary factors impacted by educational debt as savings, housing, leisure, discretionary spending, and family planning. Qualitative findings revealed impacts on themes of “personal factors” (81%), “professional factors” (62.5%), and “psychological factors” (56%) 5 years after graduation. Most negatively impacted were housing decisions, hours worked, initial job selection, and ability to save for the future, contributing to decreased mental health wellbeing with anxiety, frustration, and guilt. The majority (75%) of respondents perceived a high degree of value during and following their DPT education, though many expressed discordance between expectations and realities of practice.

**Discussion:**

Findings demonstrate that impacts of health professional educational debt in professional, personal, and psychological factors continue 5 years following degree completion, regardless of debt load. Successful implementation of this pilot methodology indicates potential for use of such extended data collection strategies. Further research is needed at the programs, profession, and/or interprofessional level to garner depth of understanding to guide interventions designed to mitigate or prevent these long-term repercussions.

## Introduction

The current generation of health professional program graduates faces a growing problem: the cost of entry-level education. Unlike some countries offering low- or no-cost college tuition, individual cost burden and correlated debt loads in the United States are rising, and the spiraling after-effects are rightly a source of acute concern raising alarms across institutions and occupations (1–5). Since 1989, graduate and undergraduate tuition has risen between 100 and 200%, contrasting with a <2% increase in wages, both adjusted for inflation (6). Even smaller salary increases have been seen among healthcare professions during the same period due to changing models of reimbursement (7). To bridge the gap between growing tuition and stagnating wages, student loans are increasingly obtained to cover educational costs (8–11). Student loans now make up the second largest source of consumer debt in the United States with evidence suggesting professional students, including those in health care programs, face particularly high burdens (12, 13).

Existing data suggests few health care graduate students can complete their education without accumulating student loans. According to information collected by the College Board 84% of medical students and 90% other health science students graduate with debt (7). Average debt loads vary among professions: in US dollars, $200,000 for physicians (2019), $183,014 for veterinarians (2019), $40,000–54,999 for graduate level nurses (2017), $114,706 for physician assistants (2017), and between $83,000–124,000 for physical therapists (14–17). However, outside of medicine neither total debt loads nor the percentage of graduates accruing debt are systematically tracked restricting understanding of the issue (18–20).

Beyond this foundational data, a small body of literature has explored the influence of debt on career trajectory and personal factors. Higher debt levels have been found to affect the selection of specialties in medicine, pursuit of residencies in pharmacy, and choice of practice areas within physical therapy (19–27). Examining the personal effects of debt on medical students, Rohlfing et al. found that relative to peers at the same institution, greater debt load was associated with delays in having children, buying a home, and marriage, as well as higher levels of callousness (insensitivity) and reduced likelihood of choosing to practice in an underserved location (27). The few sources that have examined these issues outside of medicine demonstrate debt's negative impact (7, 11, 18). Limited research indicates prioritizing salary over elements of job satisfaction or preferred practice setting may contribute to burnout and decreased professional development and higher debt loads are consistently associated with lower levels of personal wellbeing (24, 27–34). Importantly, most existing research has explored these effects within a narrow timeframe. Information is typically collected immediately after or within 1 year of graduation, restricting the durational understanding of debt's impact (7, 18, 20, 21, 29). There is little dissemination to date of feasible data collection strategies and outcomes beyond 1 year post-graduation. Further research is needed in evaluating the impacts of healthcare educational debt on career and personal choices following transition to practice.

To address current gaps in the literature, this study examines the extended effects of educational debt through a mixed methods approach, contributing quantitative data, narrative contextualization, and a sample methodology to the small existing body of evidence. The primary study objective was to examine health professional graduates' perceptions of the personal, professional, and psychological impacts of educational debt 5 years following degree completion. The secondary study objective aimed to evaluate a simple methodological approach for data collection and analysis of graduates' perceptions. Recognizing educational debt as a broad problem for all health professions, this study pilots a data collection approach for graduates from one health profession, with the design intended to be applicable for any educational program.

## Materials and Methods

This study utilized a cross-sectional, mixed methods design. The sample population included one cohort of 50 Doctor of Physical Therapy (DPT) Program graduates 5 years following DPT degree completion from the author institution, class of 2014, with data collection in fall 2019. IRB approval (#STUDY00006956) was obtained prior to data collection. Eligible individuals were sent a personalized email template inviting them to participate in a faculty-led student research project. Participants completed a primarily quantitative online survey regarding physical therapist educational debt, debt repayment strategies, impact on personal factors, non-education debt, and perceived value of their health professions education. Those who opted in and consented to survey participation received a $5 Amazon e-gift card.

Survey participants were then invited to share their contact information to engage in a 15-min interview by phone. This duration was selected out of respect for the additional time invested to complete the 10-min online survey. Interview questions were crafted to gain insight into topics of interest that may be poorly captured if included in the online survey, including alumni decision-making in selecting their first position, financial impacts of debt across professional and personal life, experiences with financial-related burnout in work, perceptions of financial literacy education during PT school, and long-term (5-year) perceptions of the value of their DPT education. Structured focused interviews (35) were conducted by student researchers using a standardized script to promote consistent phrasing of the greeting, question language, follow up question language, and closing remarks. This approach was also selected to mitigate potential bias given that data collection was performed by second year students accruing DPT educational debt. To further address this potential limitation, the faculty researcher led a discussion in bias and reflexivity in advance of interviews to prepare the students to separate their personal reactions from the interview process.

Informed consent was obtained by all participants prior to data collection. Quantitative survey questions were modeled after the physician Graduate Questionnaire (GQ), mirrored existing questions from the program exit survey, and included standardized scales such as the Oldenburg Burnout Inventory (21, 32, 33, 36–38). Topics included debt-to-income ratio, educational debt repayment strategies, educational debt's impact on personal factors, non-education debt, and perceived value of DPT education. Survey responses were submitted to a secure, online data management research platform. The phone interview was developed by study personnel to offer greater narrative depth to the topics covered in the online survey. Interview topics included factors in job choice, financial and personal decisions impacted by educational debt, burnout in clinical practice, and perceived value of their DPT education. Each researcher conducting the interview entered annotated responses directly into the secure online data management research platform noted above.

Quantitative analysis of aggregate online survey question responses included descriptive statistics for participant demographics, salary, and educational debt and correlational analyses (Spearman Rho, *p* < 0.05) to explore the relationships between each of (educational debt, monthly debt payment/salary ratio, monthly debt payment) with each of (number of life factors impacted by debt, age, burnout score).

Participant phone interview responses were de-identified prior to analysis. Qualitative analysis began with open coding, the research team (4 graduate DPT students and 1 DPT faculty) individually evaluating all participants' responses to iteratively develop a unique set of codes by which to represent those responses. The research team then established a shared set of codes, with disagreements resolved by consensus. Each researcher revisited participant's responses, assigning the new codes, continuing until the team agreed on the coding selection for each participant response. The next phase of axial coding required the collective research team to analyze the relationships across the initial open coding responses and reassemble the information to develop overarching themes in responses. Disagreements between research team members were discussed and resolved via majority vote. Finally, participant response rates for each code and theme were calculated by tallying the frequencies in aggregate.

## Results

### Quantitative

Of 50 eligible alumni, 17 participants completed the quantitative online survey (34% response rate). The majority of respondents resided in the state of the author institution and were between 30 and 34 years of age. Refer to Table 1 for participant demographics. The most frequently reported annual salary range was $80,000–89,000/year. Educational debt levels were more evenly distributed between $0–>$140,000. Both annual salary and educational debt data are found in Table 2. The personal factors most commonly cited as impacted by educational debt are found in Table 3. The factors cited by more than half of interviewees were savings (88.2%), housing (82.4%), leisure (76.5%), discretionary spending (70.6%), and family planning (64.7%). Correlational analyses, found in Table 4, demonstrated no statistical significance (Spearman's Rho, *p* < 0.05) when comparing each of (educational debt, monthly debt payment/salary ratio, monthly debt payment) with each of (number of life factors impacted by debt, age, burnout score).

**Table 1:** Participant demographics.

**Demographics**	**Respondents**
	**(*n* = 17)**
**Age (years)**	
<30	1
30–34	12
35–39	1
40–44	3
**Gender**	
Female	12
Male	5
**State of residence**	
State of author institution	13
Adjacent state	3
Non-adjacent state	1
**Population size of residence**	
>500,000	9
50,000–500,000	3
<50,000	5
**Household income**	
1 income	1
2 income, respondent's is higher	6
2 income, respondent's is lower	8
2 income, <10% difference	2

**Table 2:** Participant salary and total educational debt.

**Salary and debt**	**Respondents**
**($)**	**(*n* = 17)**
**Annual salary ($)**	
60,000–69,999	2
70,000–79,999	4
80,000–89,999	8
90,000–99,999	2
100,000–109,999	0
>110,000	1
**Total educational debt ($)**	
0	3
0–20,000	1
20,000–40,000	0
40,000–60,000	2
60,000–80,000	1
80,000–100,000	3
100,000–120,000	3
120,000–140,000	1
>140,000	3

**Table 3:** Personal factors impacted by total educational debt.

**Personal factors impacted by total educational debt**	**Respondents**
**(Choose all that apply)**	**(*n* = 17)**
Savings	15
Housing	14
Leisure	13
Discretionary spending	12
Family planning	11
Transportation	6
Other loans	5
Other	1

**Table 4:** Correlational comparison between each of (educational debt, monthly debt payment/salary ratio, and monthly debt payment) with each of (number of life factors impacted by debt, age, and burnout score).

**Correlational comparison (Spearman's ρ)**	**ρ (rho)**	***p*-value (CI 95%)**
**Moderate positive correlations**		
Higher educational debt vs. younger age	0.45	0.07
Higher educational debt vs. higher # of life factors affected by debt	0.37	0.14
**Weak positive correlations**		
Higher educational debt vs. higher total burnout score	0.29	0.26
Higher monthly debt payment/salary ratio vs. younger age	0.29	0.25
**Negligible correlations**		
Higher monthly debt payment vs. younger age	0	1
Higher monthly debt payment/salary ratio vs. higher total burnout score	−0.06	0.83
**Weak negative correlations**		
Higher monthly debt payment/salary ratio vs. higher # of life factors affected by debt	−0.29	0.26
Higher monthly debt payment vs. higher # of life factors affected by debt vs.	−0.3	0.23
**Moderate negative correlations**		
Higher monthly debt payment vs. higher total burnout score	−0.4	0.11

*Strength of correlation coefficients determined using Dancey and Reidy's interpretation (39)*.

### Qualitative

Of the 17 participants completing the survey, 16 also engaged in the subsequent qualitative phone interview (32% response rate). Though the invitation to participate suggested a 15-min duration, interviews typically extended to 25–30 min at the discretion of the respondent due to engagement in meaningful conversation. Through open and axial coding, participant responses within the phone interviews regarding the impact of educational debt were categorized into 6 themes: “personal factors” affected by debt, “personal factors” unaffected by debt, “professional factors” affected by debt, “professional factors” unaffected by debt, “psychological factors” affected by debt, and “psychological factors” unaffected by debt.

Personal impact related to life-style factors and spending, not directly related to participants' jobs. Professional impact included any statement directly in reference to participants' past or present job and/or career path. Psychological impact encompassed any participant statements that referenced emotional state, mental health, or behavior. “Other factors” served as an additional categorization to capture remaining concepts not otherwise incorporated.

**Figure 1:**
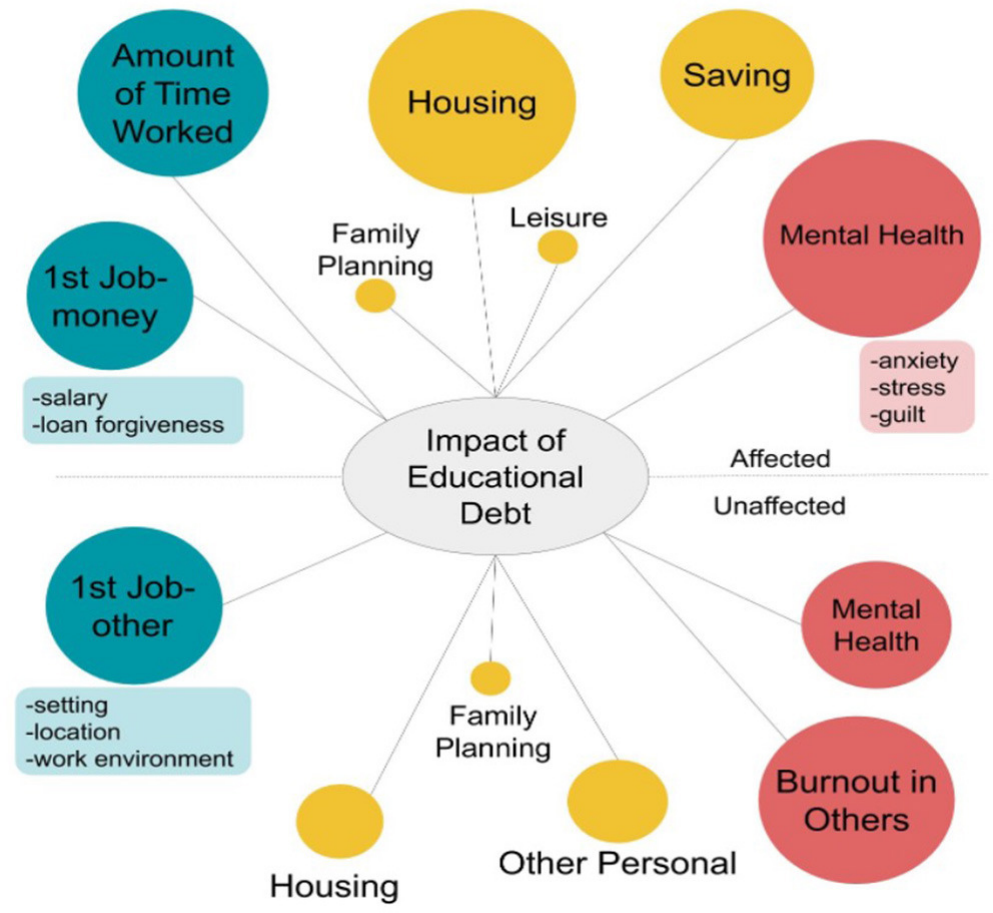
Qualitative themes identified as affected or unaffected by DPT educational debt. The frequency of participants reporting each factor is represented by the size of bubbles, with larger bubbles indicating more responses.

Qualitative themes and sample participant responses are presented in Table 5. Qualitative results in pictorial form are presented in Figure 1. Note that the number of respondents reporting each factor is represented by the size of the bubbles, with larger bubbles indicating a greater volume of responses. Factors reported as impacted by educational debt are represented in the top half of the figure, while factors unaffected by educational debt are represented in the bottom half of the figure. Findings are color-coded to indicate the associated category of professional, personal, and psychological factors, further described below.

**Table 5:** Qualitative themes and example responses.

**Qualitative themes**	**Affected**	**Unaffected**	**Example responses**
1. Impact on Personal	81%	19%	All: *Educational debt has affected buying a house, a car, every big purchase*.
Factors			Housing: *My student loan is more than the cost of my first house*.
			Loans: *You can get a home loan for 3% interest, it's crazy that we charge students 7%*.
			Budgeting: *Travel has been put on hold, I'm more conscious on budgeting. I would like to move but can't save money because of loan payments. Leisure hobbies have been a low priority because it's important to try to maintain a budget*.
			Family: *Having children required costly medical intervention. I had to rely on my family to help financially because of so much debt. And even though I have good credit, my family had to co-sign when I took out housing or medical loans due to astronomical debt*.
			Saving: *I was not able to save for college for my son, I could not contribute as much as I wanted*.
2. Impact on Professional Factors	62.5%	37.5%	Salary: *It's harder for people who go straight from undergrad to DPT because they don't often have a spouse, kids, house, and haven't lived in the “real world”, then realize salary doesn't go as far as you think, especially with debt burden. You may know the number of the salary you will be earning, but not what that means in terms of spending*.
			Work Time: *I would work part time if I didn't have to worry about paying off loans and the public service loan forgiveness requirements*.
			Job choice: *I had to do the income-based repayment plan, so I'm locked into working for a non-profit for 10 years. Which is fine, I'm ok with it, but there's no other way I could afford to pay off my loans at all*.
			Benefits: *I can't reduce to part time because of feeling like I have to make minimum loan payments and need full-time benefits because I have to care of my family and provide*.
3. Impact on Psychological	56%	44%	Guilt: *My debt makes me feel really bad about myself – it's a looming aspect, there is always guilt*.
Factors			Stress: *A coworker mentioned she paid off her loans, and it made me feel really bad about myself*.
			Anxiety: *My loans are a heavy weight*.
			Burnout: *Burnout is real, especially because you have to work extra hours to make enough for loan repayments*.
4. Other Factors			Love of the PT profession: *I think I liked being blissfully ignorant about debt burden because I was and still am passionate about what I do*.
			Love of the PT profession: *No one does what we do*.
			Perceived value of the PT profession: *My perception going in was that we were changing scope of practice with the DPT and would not have to work so directly under physicians… Still a long way to go to get to the autonomy of direct access DPT care*.
			Perceived value of the PT profession: *Pay is low for the help we give*.
			Decision to enter the PT profession: *I think if I knew then what I know now about debt, I would have chosen a different path like PTA, which is really hard for me to say*.

Responses regarding the impact of educational debt on “personal factors” were categorized as affected for 81% of participants and unaffected for the remaining 19% of participants. Eight participants cited housing decisions as affected by debt, five cited savings for the future, and three each cited leisure activities and family planning. Despite the high number of respondents citing home buying, most were still able to purchase a home albeit on a delayed timeline or with a smaller mortgage. Several participants drew direct comparisons between student and home loans. A number of respondents reflected, for themselves and other graduates, on the differences in perceived personal burden depending on whether one had a joint income or sole income household. Some also commented on relationships in which both individuals have high student debt, exacerbating the impacts on personal factors further. Sample participant responses regarding impact on personal factors are presented in Row 1 (Table 5).

Responses regarding the impact of educational debt on “professional factors” were categorized as affected for 62.5% of participants and unaffected for the remaining 37.5% of participants. For those professionally affected, job choice was dictated primarily by salary, opportunity for loan forgiveness, or access to benefits. Job setting selection within this group was less focused on specialization, and typically included skilled nursing facilities, travel physical therapy, and home health. For those professionally unaffected, job choice was dictated by factors other than money (practice setting, location, and available mentorship). Job choice within this group tended toward greater specialization, including pediatrics, neurologic, opportunities within Veterans Affairs (VA) organizations, or specialty gyms. Amount of time worked was directly impacted by educational debt for participants, with some needing to work more hours, while others desired to reduce hours but felt they could not for financial reasons. These additional jobs were cited as a source of increased stress and negative emotional impact. Three participants stated an interest in working part-time, but none were able to reduce their hours due to loan repayment demands and the associated loss of full-time benefits. Sample participant responses regarding impact on professional factors are presented in Row 2 (Table 5).

Responses regarding the impact of educational debt on “psychological factors” were categorized as affected for 56% of participants and unaffected for the remaining 44% of participants. Comments frequently referenced anxiety, stress, and dissatisfaction in relation to debt. When asked about the presence or absence of burnout in practice, 12 respondents denied personal burnout but cited observing burnout in other physical therapists, though this was attributed to practice demands rather than educational debt. Those reporting feelings of burnout attributed them to limited flexibility in decision-making on hours worked or job selection. Sample participant responses regarding impact on psychological factors are presented in Row 3 (Table 5).

The “other factors” category included responses offered by participants without prompting, not directly asked through standardized interview questions. These included comments regarding perceived value of the physical therapy profession, love of the profession, and decision to enter the profession. Over half shared a continued love of the physical therapy profession 5 years following graduation, though 19% stated they may have considered other career options specifically due to the educational debt incurred. In two cases, training as a physical therapist assistant was mentioned as a less costly alternative. Sample participant responses regarding other factors are presented in Row 4 (Table 5).

A near universal theme among participants was the desire to have addressed student loan burden sooner. One noted, *I wish it was something that we would have talked about earlier so we could start thinking and planning*. Nearly 90% of respondents felt ill-prepared by their academic institutions or lenders to deal with their debt loads, one commenting, *I felt like I was blindly taking out loans in school*. Many reflected they would have made different borrowing choices if they had more information prior to or during their DPT education, though a number commented by that point loans were already obtained so may have been too late.

Despite incurring educational debt, many respondents reported that the financial investment to earn a DPT degree was worthwhile. The majority (75%) of respondents perceived a high degree of perceived value during and following their DPT education. Some directly commented on sustained or increased perceived value regarding their DPT education 5 years following degree completion. One specified that this is due in part to the high stability and reasonable salary earned as a physical therapist. Another commented that their confidence in practice is due in part to receiving a high-quality education. Many respondents demonstrated clear pride in the PT profession and in being a PT.

However, unprompted, nearly two thirds of participants stated that they felt physical therapists are undervalued in practice, citing lower than expected pay and a lack of autonomy. Most believed the profession lacked an autonomy of practice commensurate with the DPT education level. One reflected, *going into the program, I thought I would have more autonomy. Now I realize the medical field is highly determined by physicians and insurance*. Pay was frequently tied to comments about perceived value of the PT profession and benefits offered.

## Discussion

This study adds to the growing body of literature exploring the impacts of health professional educational debt, unique in its data collection 5 years following graduation as opposed to the more common new graduate or 1 year post-graduate perspectives. Findings support and expand understanding of the extent to which educational debt acquired to pursue the DPT degree impacts professional, personal, and psychological factors beyond the first year after degree completion. This line of research has implications for all health professional programs and professional associations in their efforts to both better understand and mitigate negative effects of educational debt.

The lack of correlation found quantitatively between the amount of educational debt (operationalized in three ways: total educational debt, amount of monthly debt payment, and monthly debt payment to salary ratio) and the degree of impact (operationalized as number of life factors impacted by debt and burnout score) indicates that the negative repercussions of incurred educational debt over time are not limited only to those with significantly high debt load. These findings suggest instead that health professions alumni perceive impacts of their educational debt on their lives, regardless of the amount of debt incurred. Interventions from health professions education programs should therefore be tailored for all prospective and/or current students, regardless of amount of educational debt.

Qualitative findings demonstrate that one potential mitigating factor may be to increase financial education provided to students (1, 18, 29). Questions remain around who is most responsible and what timing is most effective for providing information related to personal financial management and decision-making. Though respondents in this study frequently reported a lack of knowledge and practical skills in managing educational costs as students, opinions varied as to whether responsibility for this education fell to students and their families, undergraduate universities, professional programs, or external financial institutions. Because many students enter their health professions program with prior undergraduate debt and incur more significant debt during their graduate education, it may be most beneficial to provide financial training prior to matriculation (1, 22). However, participants in this study frequently cited the desire to learn cost saving strategies during DPT education, echoing findings by Stepp et al. who recommended incorporating financial literacy into DPT curriculum as a useful way to reduce the impacts of educational debt on graduates (20).

Another mitigating factor for health professions programs may be to increase transparency of total degree cost and projected debt to income ratios to better inform potential applicants of their financial commitment. As a positive example, since data collection for this study, the American Physical Therapy Association published a position statement promoting financial transparency and financial literacy for applicants and students (40). Collaborations across multiple health professions educator associations may have even greater capacity to develop interventions and outcomes evaluation processes for the impacts on all health professions students and graduates.

The mixed methods, cross-sectional design in this study promoted procurement of the desired depth of response data. Quantitative results offered baseline contextual information regarding program graduates' debt and income status, current wellbeing, and perceptions of educational value, while qualitative results provided additional depth regarding a variety of perceived impacts of education debt. Successful implementation of this pilot methodology indicates potential for use of similar extended data collection strategies.

While the scope of this pilot involved a small sample size from one health profession program, the novel data collection approach demonstrates promise for generalizability in exploring the broader, longer-term effects of educational debt by any professional program. The proposed categorization of resultant impacts on personal, psychological, and professional factors provides a consistent framework for qualitative data analysis and reporting to promote comparisons over time, across programs, and across health professions. Multi-institution collection of alumni perspectives at graduation and expanded to five or more years post-graduation will provide insights to programs and to the collective professional organizations regarding the impacts of educational debt beyond solely bank accounts. This data may then serve as a catalyst for actionable steps to better inform, support, and prepare current and prospective students, and to design data-informed efforts to proactively mitigate or prevent the long-term repercussions of educational debt.

In the absence of centralized national data collection systems for allied health profession programs, it is imperative that programs begin to systematically gather information on educational debt and its impact along a shared timeline. Further research is needed at the programs, profession, and/or interprofessional level to generate greater depth of understanding, allowing more comprehensive analysis to inform state and national professional initiatives in promoting wellbeing in graduate transition to practice. Building upon this study, educational researchers can collaboratively develop a more comprehensive understanding of the interplay between educational debt and the lives of program graduates. Though dissemination of such efforts are beginning to appear in the literature, far more research is needed to develop approaches to reduce or prevent the long-term repercussions, ultimately enhancing the wellbeing of the next generation of health professionals.

## Data Availability Statement

The datasets presented in this article are not readily available because they remain in protected files. Requests to access the datasets should be directed to Sara North, snorth@umn.edu.

## Ethics Statement

The studies involving human participants were reviewed and approved by University of Minnesota Institutional Review Board. The patients/participants provided their written informed consent to participate in this study.

## Author Contributions

All authors listed have made a substantial, direct, and intellectual contribution to the work and approved it for publication.

## Conflict of Interest

The authors declare that the research was conducted in the absence of any commercial or financial relationships that could be construed as a potential conflict of interest.

## Publisher's Note

All claims expressed in this article are solely those of the authors and do not necessarily represent those of their affiliated organizations, or those of the publisher, the editors and the reviewers. Any product that may be evaluated in this article, or claim that may be made by its manufacturer, is not guaranteed or endorsed by the publisher.
